# Echo and the Failure of Knowing in Judith Fox’s Photographic Project *I Still Do: Loving and Living with Alzheimer’s*

**DOI:** 10.1007/s10912-018-9516-2

**Published:** 2018-04-26

**Authors:** Agnese Sile

**Affiliations:** Edinburgh College of Art, University of Edinburgh, ECA Main Building and Hunter Building, 74 Lauriston Pl, Edinburgh, EH3 9DF UK

**Keywords:** Alzheimer’s, Photographic essay, Visual illness narrative, Couple, Relationship

## Abstract

In relationships ‘I’ and ‘you’ become ‘we’; despite individual differences, couples obtain an interdependent identity due to their shared interactions. During a serious illness, biological and biographical disruptions can put any reciprocal relationship under strain. Through intermedial analysis of Judith Fox’s photographic project, *I Still Do: Loving and Living with Alzheimer’s* (2009), I will explore ways the couple make sense of illness, how illness is communicated through text and image and also to identify the limits of representation. Here the photographs, I argue, solidify their relationship and echo the stuck-in-the-present state of mind brought on by Alzheimer’s.

## Introduction

In relationships ‘I’ and ‘you’ become ‘we’; despite individual differences, couples obtain an interdependent identity due to their shared interactions. In a paper ‘Experiences of Sexuality and Intimacy in Terminal Illness: a Phenomenological Study,’ Bridget Taylor argues that ‘When someone is living with a life-limiting illness, their coupled relationship is also dying’ (2014, 438). The constant trial of living, despite biological and biographical disruptions, puts any reciprocal relationship under strain. Couples often must adjust their attitudes and actions to maintain a sense of intimacy. A future planned together, the expectations and predictability of the relationship, are called into question.

In this paper, I will analyse American photographer Judith Fox’s project, *I Still Do: Loving and Living with Alzheimer’s* (2009), which depicts the devastation of Alzheimer’s that struck the artist’s husband, Dr. Edmund Ackell (addressed as Ed in the project). Through intermedial analysis, I will aim to investigate what kind of knowledge is contained within this photographic project, how the photographs and the accompanying text construct experiences and articulate private expressions of illness and what are the limitations of these representations. My intention and focus are on identifying and illuminating the intimate space that opens between the photographer, the photographed person and the viewer as well as the dynamics of collaboration between those involved in the photographic act.

Sequential photographs, which are not necessarily represented in linear order, connect relational, imaginative, sensual and emotional elements and experiences. Photographs are ‘structured and structuring spaces’ (Bell 2002, 9) within which the photographer, the photographed subject and the viewer can engage with each other and through this process create new meanings. This method of communication is multi-sensory and less limited than traditional verbal communication, which is often difficult or impossible for people with neurodegenerative conditions, such as dementia and Alzheimer’s. Although, the photographic essay, *I Still Do: Loving and Living with Alzheimer’s* is mediated by Fox who collaborated with her husband, her skills as a photographers are used ‘in the service of conferring on the sufferer a dignity’ (Radley 2002, 16). Fox not only expresses her own grief and suffering at and by seeing the suffering but also performs a function of speaking for her husband who cannot do so because his *authorial* voice is too weak, fragmented and incoherent (Hydén 2008). The photographic act that makes an image possible reverberates amongst those who took part in it and those who view and read these works, long after that act took place.

## *I still do*

*I Still Do: Loving and Living with Alzheimer’s*, which was published in book format in 2009, is a photo essay by American entrepreneur turned professional photographer Judith Fox. Her husband, who was a surgeon and a president emeritus of Virginia Commonwealth University, was diagnosed three years after their wedding in 1998. Fox’s and Edmund’s planned togetherness, their future-directed life, was interrupted by a devastating illness which has no cure. The idea to create a photographic record and publication of the experience grew out of protest against misinformation, stigma and shame, and ‘to clear the cloud of fear and whispers’ that surrounds Alzheimer’s disease (Fox 2009, 124).

The photographic essay consists of forty-seven colour stills and forty-two pages with short texts written by Fox that accompany the photographs. She started taking photographs of Edmund three years after his diagnoses in 2001 and finished in 2009, when due to severe visual agnosia he could no longer see the images and her continued photographic practice of him ‘would be compromising his dignity’ (Fox 2010). This statement shows Fox’s commitment to sustain a constructive verbal and visual dialogue between them. When Edmund is no longer able to actively participate in this exchange and creative collaboration, she ends the photographic project.

Fox describes her and her husband’s mutual agreement to record their experiences. Toward the middle of the book, she writes: ‘I told Ed that some of the photographs I took of him saw straight through to his soul and asked if he minded being that exposed. He said “No. You can show my soul; just don’t show my penis”’ (65). Fox complies with this agreement and does not break their mutual trust. In *I Still Do*, conversations, exchange of gazes, and moments of togetherness between Fox and her husband are externalised and become the basis of continuous dialogue between viewers and the participants of the photographic act. As the story unfolds, it discloses a cluster of emotions and themes such as connection, fusion, disconnection, fear arising from the possibility of losing the other, separation and tension. The relationship here is not static and stable but rather a dynamic experience, interaction and exchange that occurs between individuals. Photography provides an intimate space that can be accessed not only through the visual and therefore graspable but more importantly through tactile and auditory senses in which the relationship can be played out.

**Fig. 1:**
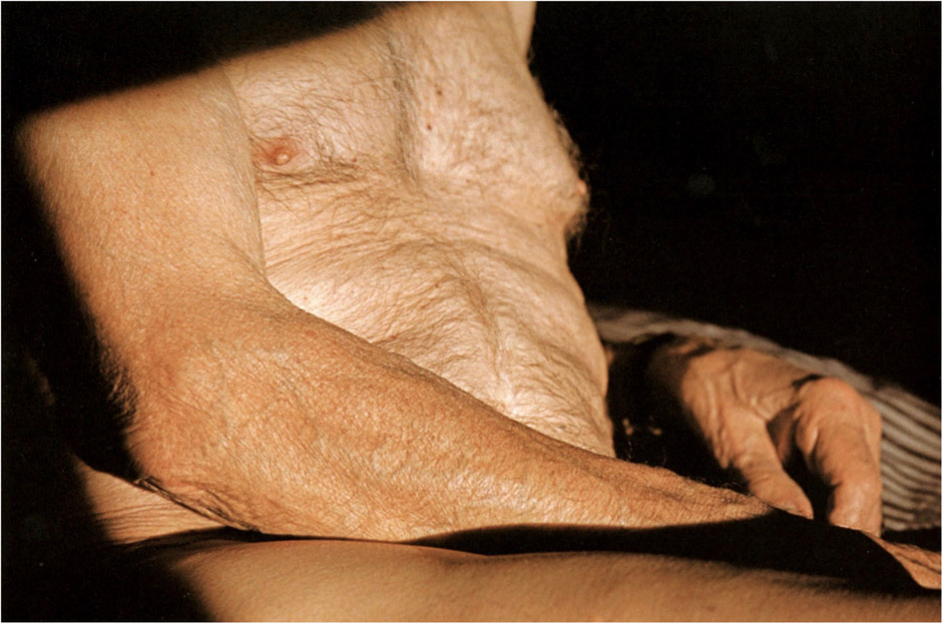
Untitled image of Edmund (Fox 2009, 64)

There are close-ups of Edmund’s face, feet, hands, bare torso and back that fill up the photographic frame. The extreme proximity of Edmund’s body and skin expresses the wish to be with him to nurture, protect and soothe him (Fig. 1). Fox writes: ‘When I watch Ed through my camera lens, despite the distance of several feet between us, I feel as though I am caressing him’ (124). She continues, writing that her camera ‘isn’t an obstruction, it’s another way of touching him’ (124). The aesthetics of the photographs recall Agnes Varda’s representation of her late husband, Jacques Demy in *The Beaches of Agnès* (2009). In an interview, Varda says: ‘The film is with Jacques who is sick’ (cited in Wilson 2012, 24). Similarly for Fox, the photographs are not about Edmund who is sick but the possibility of being with him. The photographic act, an activity in which they both participate, allows Fox not to arrest or resist the passing of time but to be in the moment with her husband: ‘I photograph Ed to remember him, to celebrate him, and to keep him close as he’s leaving’ (9). Edmund in these photographs is Fox’s ‘husband,’ ‘friend,’ ‘lover’ and ‘muse,’ and he is also a creator of his photographs (9). The project began as a cooperation between partners, out of Fox’s need to capture and behold moments of togetherness in a photographic image, and Edmund gave his approval to make a photographic record of him. However, as I will illustrate further, the project developed into a collaboration in which ‘self and other are built and connected’ (John-Steiner 2006, 5). The project challenges binaries associated with traditional gender roles as well as relationships associated with portrait photography: photographer-active, sitter-passive, object/subject, carer/cared-for. The interdependence and emotional and cognitive dependance in the project are carefully balanced and negotiated. *I Still Do* exists as a mutual gift—a gift from Fox to Edmund, as a ‘celebration’ of his existence in her life and from Edmund to Fox as a space where she can revisit and re-live their moments of togetherness when he is no longer there. The memory of Edmund survives, creating the paradox that despite biological death, a person can still exist. He exists and matters to those with whom his life has been interconnected and also to the readers and observers who have been touched by the images and text. Fox’s photographs and text provide an illusion of continuity over time and space.

## The stigma of ageing

In *I Still Do*, Fox not only raises awareness of Alzheimer’s but also creates a counter-narrative to existing stereotypes of ageing, mature love and eroticism. Jeff Hearn in *Imaging the Aging of Men* states that ‘[o]lder men are constructed as pre-death. They are relatively redundant, even invisible’ (1995, 113). In America’s youth-oriented culture, which despises dependence and decay, the elderly are mostly visible only in daunting numbers of statistics (Kashi 2005, 195). News stories often identify and comment, directly or indirectly, on the person’s age; older people who have remained active, are involved in community and have preserved their youthfulness, are commonly praised, whereas those who ‘are seen to have failed’ because of illness, accident or whose body function and structure have been impaired often evoke ‘pity, fear, disgust, condescension, and neglect’ (Featherstone and Hepworth 1995, 29-30). Hearn illustrates several recurring genres in which ageing men appear in media. Politicians, businessmen, experts and broadcasting personalities are often depicted as ageless, ‘asexual, agendered’ individuals whose power and authority are not affected by ageing (1995, 108). Another scenario which encompasses ageing men is a ‘moving on’ narrative or what Hearn calls ‘the theme of the “aging king”’ (109). Typically, these are successful sportsmen or musicians who are approaching retirement; the change in their occupational status does not involve a change in their status. These mass-produced stereotypes show a lack of concern for the problems and ‘realities’ of ageing. Age in these scenarios is constructed as ‘a maker of power, or as a reference point of power, even when power is lacking’ (Hearn 1995, 102). Positive ageing, for Mike Hepworth ‘should be an ironic acceptance of the natural ending of one’s life’ (1995, 190) and therefore a critical response to society ‘which disregards the inevitable physical decline of an ageing body’ (Fairhurst 2012, 192). As I will illustrate, in *I Still Do*, Fox reveals her husband’s ageing body, however, without romanticising Edmund’s illness. The photographs also depict beauty and communicate an acceptance of Edmund’s inevitable demise.

**Fig. 2:**
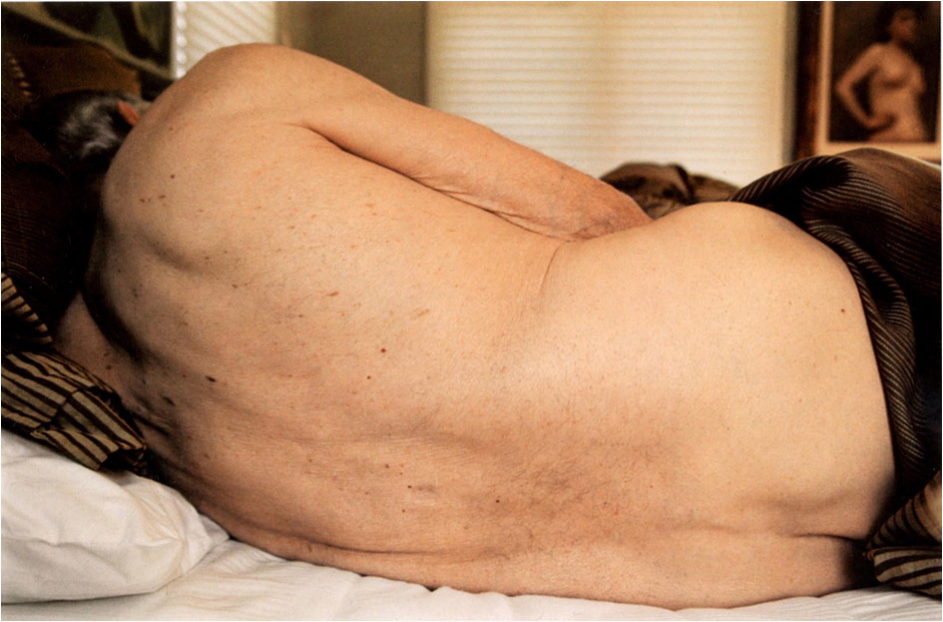
Untitled image of Edmund sleeping (Fox 2009, 83)

A photograph depicts Edmund resting in the bed (Fig. 2). His exposed back, which is turned towards the camera, stretches across the entire width of the photographic frame. The blinds are drawn, natural light is seeping through them, suggesting that it is either morning or day time. Edmund’s back is curled, his head is only partially visible in the image. The photograph allows us to observe the imperfections of his skin against the smooth textures of the bedding—small hairs growing on his back, birth marks dotted across his body, bluish veins that are faintly visible on his arm and wrinkled skin on his elbow. The image radiates warmth and affection. The viewer’s gaze follows the shape of Edmund’s back and its curved spine in a forwards and backwards movement, which recalls a physical act of cradling. As Jennifer M. Barker writes: ‘The viewer caresses by moving the eyes along an image softly and fondly, without a particular destination’ (2009, 32). The composition of the image and Edmund’s pose recollect paintings of reclining nudes in Western art such as Diego Velasquez’s *The Rokeby Venus* (c.1647-51) and Henri Matisse’s painting *Reclining Nude, Back* (1927)*.* The resemblance is somehow more emphasised by a painting of a female nude that looks back at Edmund and the viewer from the top-right corner of the photograph. Fox’s photograph depicts Edmund’s unclothed body as dynamic and sensual, yet she does not idealise it. His ageing and sick body is not meant to evoke pity and loathing; it is a site for affection and eroticism. The form of his body is also a mode of expression.

The media theorist Vilém Flusser, in his book *Gestures*, claims that ‘exhibitionism is the gesture of loving’ (2014, 48). For Flusser, gesture is ‘a movement of the body or of a tool connected to the body for which there is no satisfactory causal explanation’ (2). Gesture here is a translation of an abstract concept into a physical and material act. Precisely due to the materiality of gesture, Flusser argues that the gesture of love is doomed to failure, as it describes either sexual or mystical experiences (52). Yet what else are Edmund’s gesture of sleepy and erotic presence, and Fox’s photographic act, if not ‘an action of love and unquestioning unjudgmental loyalty, of attention and responsiveness to a beloved particular’ (Nussbaum 1992, 362)? Gesture as a movement requires a space, and the photographic act creates that space. In *Camera Lucida* Roland Barthes writes:

The presence (the dynamics) of this blind field is, I believe, what distinguishes the erotic photograph from the pornographic photograph. […] The erotic photograph, on the contrary (and this is its very condition), does not make the sexual organs into a central object; it may very well not show them at all; it takes the spectator outside its frame, and it is there that I animate this photograph and that it animates me. (2000, 57-59)

Barthes finds the *punctum* of the photograph in this ‘subtle *beyond*’ (59) the visible frame, in the space which belongs to no one. Although this image depicts intimacy between Fox and her husband, the viewers of the photograph are also invited to remake and absorb this experience, otherwise ‘without the gesture of loving, any communicative gesture is an error. Or, as it should have been called earlier, sin’ (Flusser 2014, 54).

## In between two deaths

Alzheimer’s, classified under an umbrella term of neurodegenerative disorders that covers a range of conditions and indicates loss of functions and structure of the neurons in the brain, destroys memories, changes habits and ‘leads to a reduced capacity for self-care and self-direction’ (Woods 1989, 7). As the disease progresses, so does identity become more and more interrupted. David B. Morris in *Illness and Culture in the Postmodern Age* writes: ‘Alzheimer’s disease erodes the self […], the focus of damage is less the body than the person’ (1998, 57). If personhood is understood in terms of a continuation of oneself across past, present and future—as an ‘autobiographical self’ (Damasio 2000; Ricœur 1992), then the language breakdown, loss of memory and inability to project oneself into the future that are associated with Alzheimer’s also mark ‘the death of the self’ (Eakin 1999, 46). As the neuroscientist Antonio Damasio claims: ‘Without […] autobiographical memories we would have no sense of past or future, there would be no historical continuity to our persons’ (2000, 218). Lisa Diedrich refers to existence between the demise of the self through Alzheimer’s and the loss of biological life as a space ‘between two deaths’ (2007, 116). However, the process of the undoing of the self that is associated with Alzheimer’s is also marked with finding new ways of communicating. Mark Freeman in the essay, ‘Beyond Narrative: Dementia’s Tragic Promise,’ questions and challenges narrative as a foundational concept for understanding the self. ‘It is quite possible that by moving beyond narrative, beyond the confines of storylines,’ he writes, one could potentially ‘experience something like liberation’ (2008, 175). In his account of his mother’s experience living with dementia, Freeman notes that there are moments when his mother finds enjoyment such as listening to music or being with other people. Damasio describes this in relation to ‘core self’: ‘You rise above the sea level of knowing, transiently but incessantly, as a *felt* core self, renewed again and again, thanks to anything that comes from outside the brain into its sensory machinery’ (2000, 172 [original emphasis]). The notion of the self which is ‘renewed again and again’ through the engagement with ‘the *Other*-than self’ (Freeman 2008, 180) is of importance in the analysis of *I Still Do* project.

**Fig. 3:**
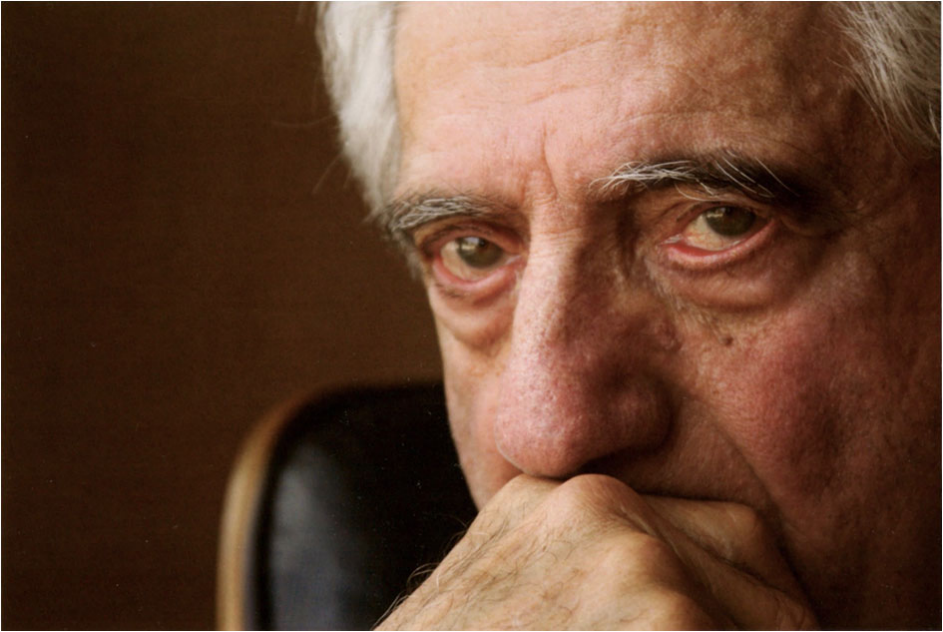
Untitled image of Edmund (Fox 2009, 11)

‘When I married Ed,’ Fox writes: ‘he was a fit, vibrant, sexy man, with a brilliant mind. Now, when I see him, part of me sees him like he is—stooped, confused, like an old man, with a disease that has taken his spirit and his memories—but most of me still sees the man I fell in love with’ (2010). Most of the images in *I Still Do* portray Edmund resting, sleeping, contemplating or confronting the gaze of the camera (Fig. 3). The stillness of composition and Edmund’s poses contrast with the text which occasionally recalls the things that Edmund used to do and the vitality that he used to possess: ‘my husband used to […]: fly a plane, perform surgery, consult worldwide, head a university and medical centres, hit four holes-in-one, and play on the same basketball team as Bob Cousy’ (Fox 2009, 15). Although Fox occasionally falls back on those memories, she does not allow herself to dwell on the past for too long. The photographic essay does not include any photographs of Edmund before his diagnosis, thus refusing the possibility of comparing and scrutinising his visible decline. Photographs depict Edmund with no visual indications of his illness; they are not dated or captioned which makes it difficult to establish how much time has passed between each image. In a way, Edmund’s illness affects and constructs Fox’s form of documenting their shared existence through Alzheimer’s disease’s ‘habit of imploding time into a present without past or future’ (Diedrich 2007, 136). The photographs capture fleeting moments. Although the photographic essay suggests a narrative, it does not follow any chronological order; there is no beginning or end but rather a middle that extends into three-dimensional space.

The word *still* appears in the title and echoes on several pages within the book: ‘In the midst of a devastating disease, there are *still* lovely moments, laughs, hands held and bodies touched’ (77 [emphasis added]); ‘we have quiet, generally uncomplicated conversations; and we tell each other “I *still* do…”’ (79 [emphasis added]); ‘Ed *still* lives at home and I’m *still* his evening caregiver’ (125 [emphasis added]), and ‘Ed, I *still* do…and I always will’ (123 [emphasis added]). To ‘be still’ means to be motionless, silent, constant, free from commotion, settled; as an adverb it signifies a now-as-before temporality (OED). ‘Still’ here signifies a refusal to collapse and let go, despite facing challenges and severe perplexity.

The constant framing with a slight variation of the size of the photographs and position of the text gives them a repetitive and therefore a reassuring rhythm, promising a control over events. Yet there is nothing still about this debilitating disease. As Fox writes: ‘Right now Ed and I are tumbling feet-over-head. No platforms in sight. A nightmare’ (74). There are also moments of hopelessness and overwhelming grief:

Who thought up the innocent-sounding euphemism ‘sun-downing’ to describe the anxious and erratic early-evening behaviour? Let's be honest, here. How about ‘howling at the moon’? How about ‘clawing at the walls’? How about the ‘twilight zone’? ‘Sun-downing’? Please. (93)

Fox addresses the lack and inappropriateness of existing language to narrate the illness experience. Medical language is either incomprehensible to non-medical people, or, as it does here, it shows a deliberate intention to lessen and devalue the anguish and distress that confusion and restlessness that are symptoms of Alzheimer’s. Instead, Fox aims to find new ways of expressing and communicating the horrors, fears, uncertainties and love that are intermingled in their relationship. The narrative and the ritual of photographic act that provide a sense of control help Fox to distance herself from the anguish and fears. For the viewers, as Arthur Frank claims: ‘the story holds us, making it safe enough - not entirely safe but compelling—to follow where it leads’ (2008, 123).

## Echo

**Fig. 4:**
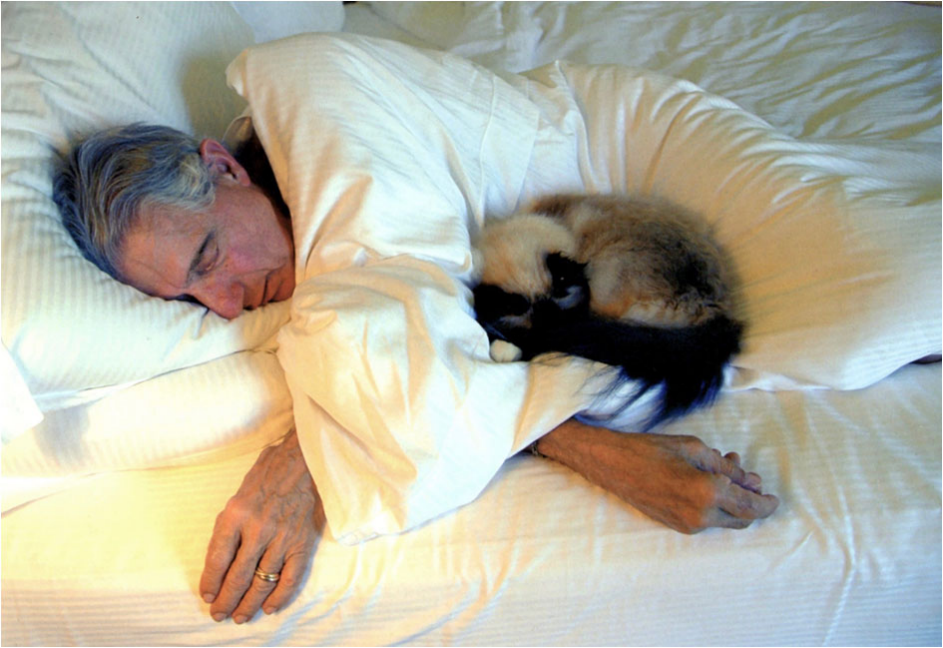
Untitled image of Edmund and a cat (Fox 2009, 25)

Edmund is predominately photographed in enclosed spaces: a bathroom, a bedroom and a courtyard. There is a sense of isolation. Only one image in the book shows other people; however, their shapes are so blurred that they appear distanced and almost erased from the photographic image. The absence of others underlines Fox’s and Edmund's emotional dependency on one another. The photographs, intimate and domestic (a vase on the table, a sheet, a cat), provide a secure space (Fig. 4), a ‘holding environment,’ to borrow Donald Winnicott’s term (1990, 47). The close framing, focus on details and fragments of Edmund’s body communicate a desire to capture and to behold. They also suggest a necessity to watch over and to be vigilant. Kelly Oliver defines vigilance as ‘both observing or keeping watch and responding to something beyond your own control’; it is a ‘movement beyond ourselves toward otherness’ (2001, 135). The time and space that couples share together make them responsive to one another. In disease such as Alzheimer’s, the person’s behaviour can become irrational and unpredictable; it is marked by hallucinations, aggressiveness and wandering. Fox writes that she is often awake during nights: ‘when he gets up to go to the bathroom, I’m there to make sure he heads in the right direction. When he jumps out of bed at three a.m. because he thinks he has a meeting to go to, I settle him down’ (87). Fox has an ethical responsibility, an obligation to be vigilant and ‘to continually open and reopen the possibility of response’ (Oliver 2001, 19). However, what is her response, since responding here does not necessarily mean answering Edmund? Jean-Luc Nancy suggests that perhaps the *obligation* to respond is more pivotal than the actual act of responding: ‘it may be that one responds to the call only by repeating the call—as night watchmen do’ (1993, 323). For Adriana Cavarero, echo, as a voice, acts like a reflecting mirror. Cavarero notes that in Ovid’s original tale, Echo ‘is transformed into an effect of resonance. She cannot speak first; but she cannot remain silent. She speaks *after*, she depends on others’ discourses and becomes merely their echo’ (2005, 166 [original emphasis]). Fox’s response to Edmund is by being there for him, and the photographic act itself testifies to that.

John Bayley in his memoir about his wife Iris Murdoch and her demise with Alzheimer’s writes:

Our mode of communication seems like underwater sonar, each bouncing pulsations off the other, and listening for an echo. The baffling at which I cannot understand what Iris is saying, or about whom or what—moments that can produce in Iris tears and anxieties […] can sometimes be dispelled by embarking on a joke parody of hopelessness, and trying to make it mutual. Both of us at a loss for words. (1998, 44)

**Fig. 5:**
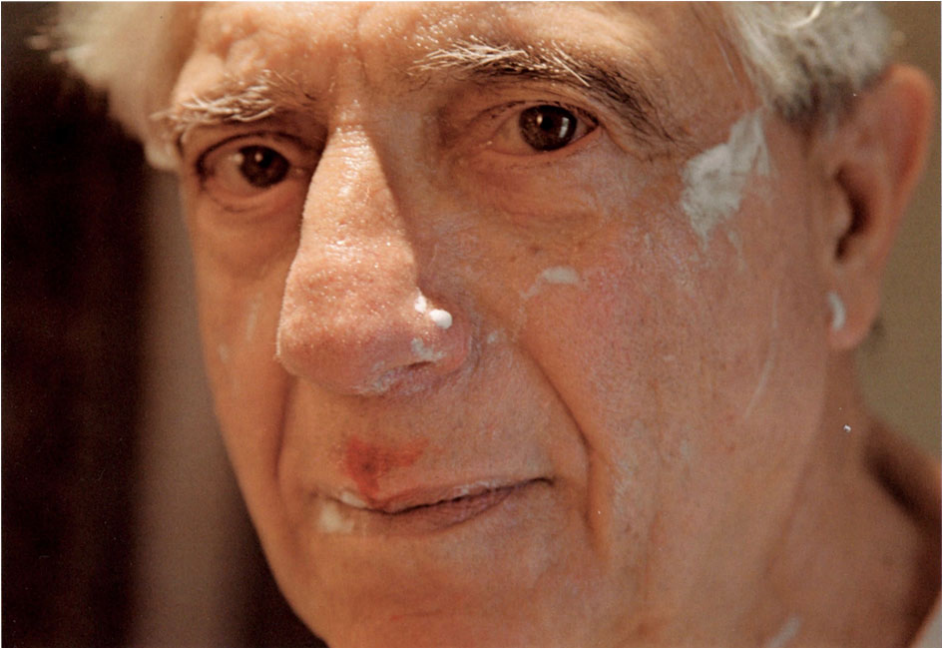
Untitled image of Edmund shaving (Fox 2009, 25)

Similarly in Fox’s photographic essay, there are plays between gazes, conversations between text and image. At the beginning of the photographic essay, Fox describes things that Edmund can still do: ‘express his love and appreciation, explain a medical issue to a lay person in such a way that they understand it, […] supply words I can’t recall, shave, and shower’ (18). Despite Edmund’s incapability, at least in his words, to perform these actions, the next three photographs depict him doing precisely that—showering and shaving. The third image shows a close-up of Edmund’s face; he is looking towards the camera, shaving foam still visible on his cheeks and lips (Fig. 5). There is a small shaving cut above his mouth through which blood is seeping. His direct gaze at the camera seeks a response and acknowledgement. The text on the opposite page seems to reassure: ‘Sometimes, “good enough” is good enough’ (24). The photographs here build a scaffolding, a safety zone in which support can be enacted and a visual reminder of what Edmund can and could do. As ‘Ed is losing his story’ (44), through the photographic act Fox reconstructs a narrative of moments and fragments of togetherness and builds a space in which their relationship can be played out.

Fox writes: ‘He wants his old life back’ (33). Later she continues: ‘No matter where Ed lives in this world, he will never again feel at home. […] Our house can no longer be counted upon to give him stable shelter and protection’ (115). Virginia Woolf (2012) associates home with a private space with the self as a centre; the home is a space in which one can protect oneself, seek intimacy and renewal, nurture wounds, unfold dreams and memories. ‘To him, “home” was a place’, Fox recollects Edmund saying: ‘where I was when I was well’ (2010). The inability to feel secure, a separation from and longing for all that is familiar, resembles homesickness: ‘Our body and our perception always solicit us to take the landscape they offer as the centre of the world. […] if I am kept far from what I love, I feel far from the centre of real life. […] Certain forms of homesickness are examples of a decentered life’ (Merleau-Ponty 2012, 299). Merleau-Ponty in *Phenomenology of Perception* questions and traces what he refers to as ‘an original spatiality’ and the ‘experience of the unreal’ (296), that which precedes familiarity with a particular thing, object or place. He finds it in the uncertainty and unfamiliarity that strike one during the night:

The night is not an object in front of me; rather, it envelopes me, it penetrates me through all of my senses, it suffocates my memories, and it all but effaces my personal identity. I am no longer withdrawn into my observation post in order to see the profiles of objects flowing by in the distance. The night is without profiles, it itself touches me and its unity is the mystical unity of the *mana.* Even cries, or a distant light, only populate it vaguely; it becomes entirely animated; it is a pure depth without planes, without surfaces, and without any distance from it to me. (296 [original emphasis])

Merleau-Ponty is not referring to blindness or the impossibility of seeing but rather to darkness that ‘envelopes me.’ His account of this experience at night helps to identify a confrontation with things, objects and places that are unconditioned by any meaning, when nothing makes sense any more. ‘Sometimes,’ he writes, ‘however, the lived distance is at once too short and too wide: the majority of events cease to count for me, whereas the nearest ones consume me. They envelope me like the night, and they rob me of individuality and freedom’ (299). However, the night for Merleau-Ponty is not the most disconcerting experience of the unreal, as one can still ‘hold onto the structures of the day’ (296). For Edmund, these structures are also failing and when he can no longer make sense of things, it is the other, in this case Fox, who helps to find ways to rebuild them.

## Failure of knowing

In between the text, images and exchange of gazes, there are tensions, anxieties and secrecies. ‘I know what’s going on in his head. He expresses his feelings when he’s awake and he talks in his sleep at night. When he’s in a room by himself he speaks his thoughts out loud. There are no secrets’ (Fox 2009, 84). However, Fox can only express and tell the experience from her point of view. At the same time, she also conceals her knowledge from Edmund: ‘I didn’t tell Ed how sick he was because he didn’t want to know. And I’ll continue to follow his lead. I’ll try not to tell him more than he wants to know. Or less’ (118). Knowing itself is thrown into question here.

**Fig. 6:**
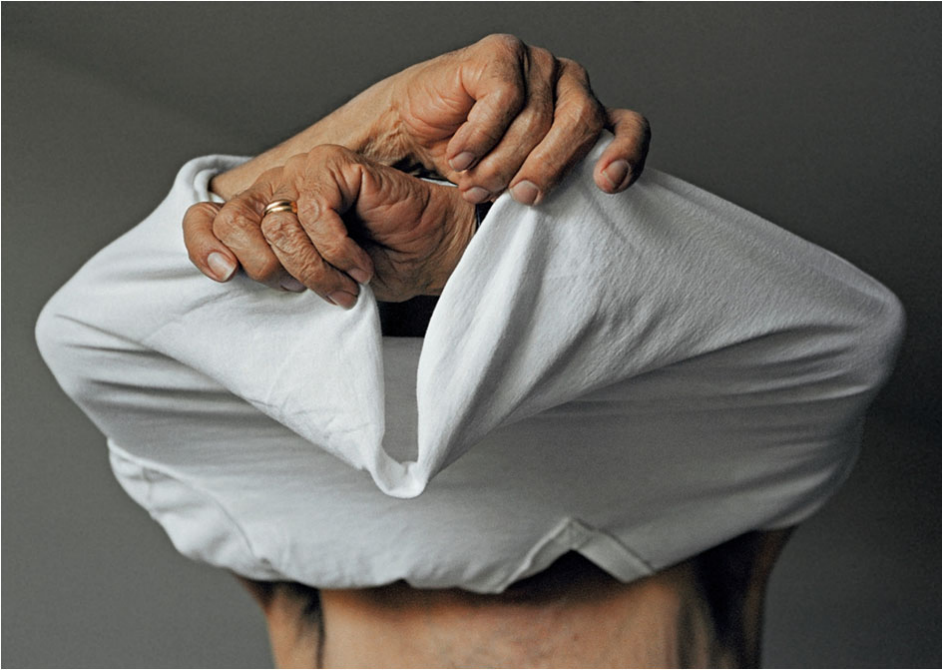
Untitled image of Edmund undressing (Fox 2009, 43)

A photograph shows Edmund at the moment of getting undressed (the same image also appears on the cover of the book) (Fig. 6). His head in covered by a white t-shirt; only his hands crossed above his head and his bare torso are exposed to view. The text on the opposite page states: ‘For several years, Ed didn’t want people to know he had Alzheimer’s. He wanted to be treated with respect and he didn’t want people to think he was “crazy”’ (42). In this context, Edmund appears as if hiding, trying to cover his face that would indicate some presence of the illness; he is tied up in the situation. Yet, the process of undressing here signifies a gesture of change, his act is not a permanent state, there is a movement towards uncovering, towards becoming visible. If the image is turned upside-down, Edmund’s pose resembles that of a sleeping bat. Edmund’s sleepiness during the day and wakefulness at night, that is described in Fox’s text and illustrated in photographs, similarly recall the nature of a bat, known for its nocturnal activities and communication abilities during the night. This association points towards the radical otherness of the other. As philosopher Thomas Nagel writes in his ‘What is It Like to Be a Bat?’: ‘anyone who has spent some time in an enclosed space with an excited bat knows what it is to encounter a fundamentally *alien* form of life’ (1974, 2 [original emphasis]). Regardless, whether Fox had intended to make her husband look like a bat or whether bats are really that ‘alien’ from humans as Nagel claims, there is a certain sense of unfamiliarity that touches Fox in her encounters with Edmund. She can only bear witness to Edmund’s changing state, and as such she is, as Oliver defines: ‘*bearing witness* to something beyond recognition that can’t be seen’ (2001, 12 [original emphasis]). This is living life as it comes, without knowing, without comprehension.

**Fig. 7:**
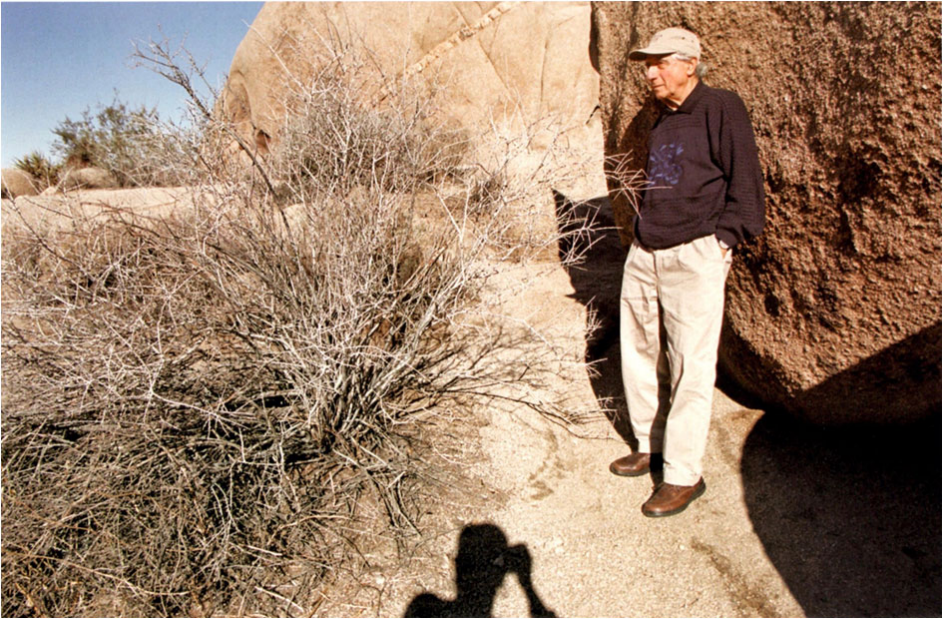
Untitled image of Edmund by a rock (Fox 2009, 61)

In one image (out of forty-seven photographs, only twelve are taken outdoors), Edmund is photographed standing against a rock in a bare landscape (Fig. 7). Strong light strikes him. Despite the sunny day, the setting looks inhospitable. The prickly leafless plant on the left and the large rocks on the right occupy most of the frame. Edmund is standing between the two; he does not look at the camera, his gaze is directed somewhere to the left. What makes this photograph so impactful is the shadow of Fox at the bottom of the image. We can see her hand at the camera, her shoulder slanted towards Edmund. Her presence is not only felt but also captured in the image. Flusser writes: ‘the man with the apparatus doesn’t move to find the best standpoint from which to photograph a fixed situation (although that may be what he thinks he’s doing). In reality, he is looking for the position that best corresponds to his intention to fix a changing situation’ (2014, 75). What Fox is capturing here is not only Edmund’s changing state but also her own changing roles—from a family friend to a wife and from a lover to a carer. Her presence in each demands a different negotiation, connection and entanglement. Fox combines her varied roles as a woman, wife, companion, carer, photographer and collaborator in a unique way, and this fluidity unsettles any stable, pre-set definition of family roles. The photographic project shows sensitivity to the other’s needs and communicates trust between them.

Taking care of someone can significantly limit one’s social life and employment status. However, the act of care affects not only those who provide care but also those who receive it. Care can deprive the other of self-sufficiency and agency. Through the photographic act—taking a picture, capturing ‘the soul of the man’ she still loves, exposing it, processing it and fixing it, Fox defies loss. Fox shows Edmund living, laughing, talking, holding her hand, reading a newspaper. She also shows what it was like to live in touch with him. Edmund’s poses and presence in the photographic frame communicate agency; as Wilson writes: ‘Capturing intimacy without exposure, the image holds the body’s agency and capacity’ (2012, 3). In a photographic image, Fox allows Edmund to articulate his agency, whilst her agency as a photographer is expressed through her decision to publish this project.

Although the subject of the image is still in the photographs, they also signify its absence. It has been there, but no longer is. For Barthes every photograph holds the possibility of demise: ‘I read at the same time: *This will be* and *this has been*; I observe with horror an anterior future of which death is at stake. By giving me the absolute past of the pose (aorist), the photograph tells me death in the future’ (2000, 97 [original emphasis]). The suspended time between past, present and future already signals inescapable loss and mourning. As Wilson notes, this ‘holding yet releasing, links love and mortality’ (2012, 2).

Sometimes Ed doesn’t remember that we’re married.

Which is something I can deal with. He never forgets that he loves me and that I love him.

Which is preferable to remembering that we’re married and forgetting that we love each other. (121)

## Conclusion

Julian Barnes in his part philosophical essay, fictionalised history and part biography on love and the loss of his wife to cancer, *Levels of Life*, writes: ‘You put together two people who have not been put together before; and sometimes the world is changed, sometimes not. They may crash and burn, or burn and crash. But sometimes, something new is made, and then the world is changed’ (2014, 31). Barnes points to interactions between partners as dynamic rather than fixed. The interdependent identity of couples is subject to growth, changes, alterations and adjustments. At the same time ‘[e]very love story is a potential grief story. If not at first, then later. If not for one, then for the other. Sometimes for both’ (2014, 36-37). In *I Still Do*, photography performs a several functions—to crystallise the trace of loss, to extend the moments of togetherness and also to provide support to those left behind after the death of the other. The photographic act in this project, I argue, gestures love and care towards the other, and the photographic image solidifies this gesture. The photographs and the accompanying text attempts to build a dialogue, gesture love and explore the echo in between the relation with the other. Photography, whilst also pointing to something that is left outside the frame, provides a space where the relationship between Fox and her husband can be played out; it provides an opportunity for intimacy. Relationships amongst partners can be fragile and vulnerable, and vulnerability is intrinsically linked to intimacy. Vulnerability is present on both sides of the camera; the experiences of the other is a reflection of one’s own potential finitude. The loss of the other, or the loss of something within the other, results in exposure of oneself, characterised by a wide range of emotions, such as grief, guilt, anger and fear.

The photographic essay deals with illness and love, which are considered as private expressions. However, photography transfers the personal into the public, therefore seeking new forms of collective outcome. As Ariella Azoulay claims: ‘a photograph produced in the course of an encounter between photographer and photographed is created and inspired by a relation to an external eye, the eye of the spectator’ (2008, 129). Viewed through the lens of public activism, the photographic essay creates a counter-statement of visual stigma and expand the understanding of lived experience. *I Still Do* shows how connecting and disconnecting in close relationship are affected by the disembodied experience and suffering from brain disorder such as Alzheimer’s. By mimicking the symptoms associated with Alzheimer’s, the project challenges the concept of a narrative self. It breaks the silence and stigma that surrounds Alzheimer’s and reconstructs the voice of Edmund whose social interaction has been affected by disease.

The project also resists conclusion; the photographs provide continuity. The space that opens up between the image and the text, as well as in the photographic act itself—between the photographer, the subject of the photographs, and viewers—presents the possibility of dialogue that has a transformative potential. For Merleau-Ponty:

In the experience of dialogue, a common ground is constituted between me and another; my thought and his form a single fabric, my words and those of my interlocutor are called forth by the state of the discussion and are inserted into a shared operation of which neither of us is the creator. […] Our perspectives slip into each other, we coexist through a single world. (2012, 370)

Photographic essays, as I have illustrated in this paper, create time and space between individual images as well as between text and image. These spaces that open up between the images and text as well as between the photographer, their sitter and the viewer of these works are critical in connecting different agencies and engaging with an open-ended dialogue.
